# Isolated anemia in patients with large granular lymphocytic leukemia (LGLL)

**DOI:** 10.1038/s41408-022-00632-6

**Published:** 2022-02-22

**Authors:** Youssef Salama, Fang Zhao, Jennifer L. Oliveira, Ji Yuan, Dragan Jevremovic, Ronald S. Go, Wei Ding, Sameer A. Parikh, Mithun V. Shah, Paul J. Hampel, Aref Al-Kali, William G. Morice, Min Shi

**Affiliations:** 1 Emory University, Atlanta, GA USA; 2 The Center for Clinical Informatics Research and Education and Department of Pathology, Case Western Reserve University, Cleveland, OH USA; 3 Division of Hematopathology, Mayo Clinic, Rochester, MN USA; 4 Division of Hematology, Mayo Clinic, Rochester, MN USA

**Keywords:** Leukaemia, Leukaemia

## Abstract

Patients with large granular lymphocytic leukemia (LGLL) frequently present with neutropenia. When present, anemia is usually accompanied by neutropenia and/or thrombocytopenia and isolated anemia is uncommon. We evaluated a cohort of 244 LGLL patients spanning 15 years and herein report the clinicopathologic features of 34 (14%) with isolated anemia. The patients with isolated anemia showed a significantly male predominance (*p* = 0.001), a lower level of hemoglobulin (*p* < 0.0001) and higher MCV (*p* = 0.017) and were less likely to have rheumatoid arthritis (*p* = 0.023) compared to the remaining 210 patients. Of the 34 LGLL patients with isolated anemia, 13 (38%) presented with pure red cell aplasia (PRCA), markedly decreased reticulocyte count and erythroid precursors, and more transfusion-dependence when compared to non-PRCA patients. There was no other significant clinicopathologic difference between PRCA and non-PRCA patients. 32 patients were followed for a median duration of 51 months (6–199). 24 patients were treated (11/11 PRCA and 13/21 non-PRCA patients, *p* < 0.02). The overall response rate to first-line therapy was 83% [8/11 (72.7%) for PRCA, 12/13 (92.3%) for non-PRCA], including 14 showing complete response and 6 showing partial response with a median response duration of 48 months (12–129). Half of non-PRCA patients who were observed experienced progressive anemia. During follow-up, no patients developed neutropenia; however, 5/27 (18.5%) patients developed thrombocytopenia. No significant difference in overall survival was noted between PRCA and non-PRCA patients. In summary, this study demonstrates the unique features of LGLL with isolated anemia and underscores the importance of recognizing LGLL as a potential cause of isolated anemia, which may benefit from disease-specific treatment. LGLL patients with PRCA were more likely to require treatment but demonstrated similar clinicopathologic features, therapeutic responses, and overall survival compared to isolated anemia without PRCA, suggesting PRCA and non-PRCA of T-LGLL belong to a common disease spectrum.

## Introduction

Large granular lymphocytes (LGL) are a morphologically distinct lymphoid subpopulation in the peripheral blood characterized by abundant azurophilic cytoplasmic granules. Large granular lymphocytic leukemia (LGLL) is a clonal hematopoietic disorder caused by an abnormal expansion of LGLs in the peripheral blood, bone marrow, and spleen [1]. The majority of LGLL cases (approximately 85%) arise from cytotoxic T lymphocytes (T-LGLL), while the remaining cases exhibit natural killer (NK) cell phenotype and are categorized under chronic lymphoproliferative disorder of NK cells (CLPD-NK) in the 2017 World Health Organization (WHO) classification of hematolymphoid neoplasms [1].

Although the pathophysiologic mechanism of LGLL is not well-understood, immunological stimuli, possibly from viruses or tumor antigens, have been implicated [2–4]. This theory has been supported by the high rate of association between LGLL and various hematologic malignancies, namely B-cell lymphoma, multiple myeloma, myeloid neoplasms, and autoimmune diseases such as rheumatoid arthritis [5–10].

The diagnosis of LGLL is established by the detection of persistent and clonal LGLL cells. LGLL cells can be identified by their specific morphology and immunophenotype in the peripheral blood or bone marrow, or detected by immunohistochemical stains (granzyme B, perforin, and/or TIA-1) in the bone marrow, highlighting the presence of intrasinusoidal cytotoxic lymphocyte infiltrates [11]. T-cell clonality can be established by monotypic TRBC1 expression or Vβ T-cell receptor (TCR) gene repertoire analysis by flow cytometry, or TCR gene arrangement by polymerase chain reaction (PCR) or next-generation sequencing (NGS) analysis [12, 13]. NK-cell clonality can be determined by the restriction of killer-cell immunoglobulin-like receptor (KIR) [14].

Performing a proper workup is the key to the diagnosis of LGLL, which can be straightforward when patients exhibit typical clinical or hematologic presentations such as repeated infections, unexplained neutropenia, or splenomegaly. However, the diagnosis of LGLL can be challenging or missed because of the lack of the typical clinical and hematologic presentations [15–17]. While anemia is common in LGLL, it is usually accompanied by neutropenia and/or thrombocytopenia. Although rare, LGLL associated with pure red cell aplasia (PRCA) has been previously reported [18–20], however, isolated anemia without PRCA has not been systematically investigated. In this report, we characterized the clinicopathologic features of LGLL presenting with isolated anemia in our practice, compared these features between patients with and without PRCA, and further evaluated the clinical outcomes of such patients.

## Materials and methods

### Patient selection

All patients who presented to Mayo Clinic, Rochester MN from 2005 to 2019 with a diagnosis of T-LGLL or CLPD-NK were included. We performed a retrospective medical record chart review for clinical and pathological data. This study was approved by the Mayo Clinic Institutional Review Board.

At least 3 of the following 4 criteria was used to diagnose T-LGLL or CLPD-NK: (1) a distinct T-cell population with coexpression of 1 or more natural killer (NK)-cell-associated antigens (CD16, CD56, or CD57) and decreased CD2, CD5, or CD7 expression; or a distinct NK-cell population with decreased CD2, CD7, CD16, or CD56; (2) a clonal T-cell population or a clonal NK-cell population; (3) intrasinusoidal cytotoxic T-cell or NK-cell infiltrates in bone marrow, spleen, or liver; and (4) persistence of the abnormal T-cell or NK-cell population or unexplained cytopenia for more than 6 months. Bone marrow biopsies were performed to evaluate other hematological malignancies and to support an LGLL diagnosis. Corresponding flow cytometry analyses were performed on peripheral blood and/or bone marrow specimens to characterize the LGLL population.

Isolated anemia was defined as: Unexplained hemoglobin (Hb) <13 g/dL for males or <12 g/dL for females, absolute neutrophil count ≥1.5 × 10^9^/L, and platelet count ≥150 × 10^9^/L. The diagnosis of T-LGLL associated PRCA was based on the following [21, 22]: (1) Markedly decreased to absent erythroid precursors in bone marrow biopsy (2) reticulocyte <0.5%; (3) Unremarkable granulopoiesis and megakaryopoiesis; and (4) No other known causes of PRCA. Patients with markedly decreased to absent erythroid precursors but borderline reticulocyte percentage (>0.5% but <1%) were also classified as PRCA.

### Flow cytometry

T-cell and NK-cell flow cytometry was performed as previously described [13, 23, 24], on a FACSCanto II or FACSLyric flow cytometer (BD Biosciences, San Jose, CA), with antibodies to CD2, CD3, CD4, CD5, CD7, CD8, CD16, CD45, CD56, CD57, CD94, CD158a, CD158b, CD158e, NKG2A, TCR *γ*/*δ* (BD Biosciences, San Jose, CA), and TRBC1 (clone JOVI-1; Ancell Corp, Bayport, MN). The TCR Vβ repertoire kit (Beckman Coulter, Indianapolis, IN) was used to detect TCR V*β* clonality.

### Molecular testing for TCR gene rearrangement

*TCR* gene rearrangement was analyzed using standard BIOMED-2 assay (Invivoscribe, San Diego, CA). Multiplex PCR for *TCR* gene rearrangement was performed for TCR V*β* and TCR V*γ*, as described previously [25]. Standard electrophoresis visual evaluation criteria were applied for BIOMED-2 data analysis.

### *STAT3* gene mutation

*STAT3* gene mutation by Sanger sequencing was performed on genomic DNA extracted from available peripheral blood samples, as previously described [23]. Three sets of primers were designed to cover the most commonly reported *STAT3* mutations in the Src homology 2 (SH2), DNA-binding, and coiled-coil domains [26]. PCR was performed using HotStar Taq Master Mix (Qiagen, Germantown, MD). PCR products were subjected to bidirectional direct sequencing on an automatic DNA sequencer using the BigDye Terminator v1.1 Cycle Sequencing Kit (Thermo Fisher Scientific, Waltham, MA).

### Myeloid neoplasm-focused next-generation sequencing

NGS was performed on extracted DNA from bone marrow aspirate as previously described [27]. A targeted NGS includes 35 genes recurrently mutated in myeloid neoplasms: *ASXL1, BCOR, BRAF, CALR, CBL, CEBPA, CSF3R, DNMT3A, ETV6, EZH2, FLT3, GATA1, GATA2, IDH1, IDH2, JAK2, KIT, KRAS, MPL, MYD88, NOTCH1, NPM1, NRAS, PHF6, PTPN11, RUNX1, SETBP1, SF3B1, SRSF2, TERT, TET2, TP53, U2AF1, WT1*, and *ZRSR2*.

### Patient follow-up

Treatment responses were assessed after a minimum of 16 weeks of therapy. Complete response (CR), partial response (PR), and no response were defined as previously described [28]. CR was defined as the attainment of a normal Hb. PR was defined as improvement in Hb by >1 g/dL without CR or a decrease in transfusion requirements by >50% for at least 4 months. No response was defined as a lack of CR/PR.

### Statistical analysis

A *χ*^2^ test, Fisher’s exact test, two-tailed *t*-test, and Wilcoxon rank-sum/Mann-Whitney *U* test were used, as appropriate, for statistical evaluation of the results. “Log-rank (Mantel-Cox) test was performed using GraphPad Prism version 9.1.2 (226) for Windows, GraphPad Software, San Diego, California USA, www.graphpad.com”. *P* < 0.05 was considered statistically significant.

## Results

### Prevalence of isolated anemia in LGLL patients and their clinical characteristics

A total of 244 LGLL patients were identified; 34 (14%) patients presented with isolated anemia at diagnosis. Among 213 identified T-LGLL patients, 31/213 (15%) presented with isolated anemia: 12/31 (39%) with PRCA, and 19/31 (61%) without PRCA. Of 31 diagnosed CLPD-NK patients, 3/31 (10%) presented with isolated anemia: 1/3 (33%) with PRCA and 2/3 (67%) without PRCA. No significant difference in the prevalence of isolated anemia was found between T-LGLL and CLPD-NK groups (*p* = 0.588).

Compared to 210 LGLL patients without isolated anemia, 34 LGLL patients with isolated anemia showed a significantly male predominance (*p* = 0.001) and fewer were associated with rheumatoid arthritis (*p* = 0.023). They also had much lower Hb levels (*p* < 0.0001), higher MCV (*p* = 0.017) and were more transfusion-dependent (*p* < 0.0001)(Table 1). Consistent with the selection criteria in this study, LGLL patients with isolated anemia had a higher absolute neutrophil count (ANC) and higher platelet count than those without isolated anemia (Table 1). There was no significant difference between LGLL patients with and without isolated anemia for age, other malignancies, history of bone marrow or solid organ transplantation, B symptoms, splenomegaly, lymphadenopathy, absolute lymphocyte count, or absolute LGLL count.

**Table 1:** Clinical features of LGLL patients with and without isolated anemia, and LGLL patients with isolated anemia presenting with and without PRCA.

Clinical characteristics	Without isolated anemia (*n* = 210)	With isolated anemia (*n* = 34)	*P* (w/ vs. w/o isolated anemia)	Isolated anemia with PRCA (*n* = 13)	Isolated anemia without PRCA (*n* = 21)	*P* (isolated anemia w/ vs. w/o PRCA)
Sex, *n*						
Male:female	126:84	30:4	0.001	11:2	19:2	0.627
Age, median (range), y	65 (39–91)	70 (32–90)	0.124	72 (41–90)	70 (32–87)	0.810
Medical history, *n* (%)						
Rheumatoid arthritis	38 (19%)	1 (3%)	0.023	1 (8%)	0 (0%)	0.400
Hematologic malignancies	32 (16%)	10 (29%)	0.054	4 (31%)	6 (29%)	0.290
Non-hematologic malignancies	23 (11%)	6 (18%)	0.299	2 (15%)	4 (19%)	0.340
BM/PBSCT transplantation prior to LGLL diagnosis	4 (2%)	2 (6%)	0.207	1 (8%)	1 (5%)	0.480
Solid organ transplantation prior to LGLL diagnosis	6 (3%)	3 (9%)	0.123	2 (15%)	1(5%)	0.270
B symptoms, *n* (%)	17 (8%)	1 (3%)	0.483	7 (54%)	9 (43%)	0.220
Splenomegaly, *n* (%)	42 (21%)	6 (18%)	0.749	2 (15%)	2 (10%)	0.350
Lymphadenopathy, *n* (%)	3 (1%)	1 (3%)	0.464	0 (0%)	1 (5%)	0.620
Red cell transfusion dependent, *n* (%)	35 (17%)	18 (53%)	<0.0001	13 (100%)	5 (24%)	<0.0001
Hemoglobin (g/dL), median (range)	11.4 (3.5–14.8)	9.1 (5.0–12.7)	<0.0001	8.5 (5.0–9.7)	9.3 (5.2–12.7)	0.120
MCV (fl), median (range)	94.5 (72–127)	99.9 (83.5–128.8)	0.017	94.6 (83.5–111)	100.9 (84.1–128.8)	0.035
Reticulocytes (%), median (range)	1.78 (0.13–8.77)	0.93 (0.07–6.09)	0.009	0.48 (0.07–0.65)	1.23 (0.61–6.09)	0.002
Reticulocytes (×10^9^/L), median (range)	53.8 (2.5–186,620)	19.3 (1.9–211.3)	0.036	10.5 (1.9–19)	30.5 (16.9–211.3)	0.010
Erythropoietin (mIU/mL), median (range)	90 (8.5–68,200)	420 (14–2883)	0.466	853 (260–2608)	151.9 (14–2883)	0.210
Absolute Neutrophil count (×10^9^/L), median (range)	0.9 (0.0–3.8)	2.5 (1.6–11)	0.013	2.6 (1.6–11)	2.4 (1.6–5.4)	0.745
Absolute lymphocyte count (×10^9^/L), median (range)	3.0 (0.7–14.3)	4.0 (0.6–9.0)	0.078	2.6 (0.6–9.0)	4.4 (1.5–9.0)	0.316
Absolute LGLL count (×10^9^/L), median (range)	2.0 (0.5–30)	2.1 (0.5–9.8)	0.668	1.8 (0.5–6.6)	2.6 (0.5–9.8)	0.127
Platelet (×10^9^/L), median (range)	183 (20–680)	283 (113–755)	<0.0001	246 (113–610)	293 (157–755)	0.609

Among 34 LGLL patients with isolated anemia, 10 had a history of other hematological malignancies: 4 plasma cell neoplasms, 2 chronic lymphocytic leukemia (CLL), 1 each of nodal marginal zone lymphoma, diffuse large B-cell lymphoma NOS, acute myeloid leukemia, and classic Hodgkin lymphoma. At the time of LGLL diagnosis, 8 patients were in complete remission for the previous hematological malignancies, and 2 had a low level of involvement (5% of cellularity), 1 by plasma cell neoplasm and 1 by CLL.

LGLL patients with isolated anemia did not have known chemical/toxic/heavy alcohol exposure. An extensive workup was performed to rule out other potential causes of anemia. No deficiencies of iron, B_12_ and/or folate were detected in all 34 patients. 33 patients had at least one bone marrow biopsy that showed no evidence of myelodysplastic syndrome or other myeloid neoplasms by thorough morphologic and cytogenetic evaluation. An NGS myeloid neoplasm panel was performed on two available patients and revealed no pathogenic mutations. No definitive hemolysis was found based on absolute reticulocyte count, LDH, bilirubin, and haptoglobin levels. Six patients had chronic kidney disease; however, the stable and mildly decreased glomerular filtration rate (GFR) did not explain the worsening moderate to severe anemia (median Hb level was 8.5 g/dL, range 6.8–9.6 g/dL). In total, 4 of 6 patients with splenomegaly underwent splenectomy with no resolution of anemia. No parvoviral infection or thymoma was found in patients with PRCA.

### Clinical and pathologic features of LGLL patients with isolated anemia presenting with and without PRCA

Of all 34 patients, 18 (53%) patients required transfusion, and patients with PRCA were more transfusion-dependent (100%) than patients without PRCA (24%) (*p* < 0.0001) (Table 1). Patients with PRCA also showed significantly lower MCV than non-PRCA patients (*p* = 0.035): PRCA patients usually presented with normocytic anemia while non-PRCA patients with macrocytic anemia. This finding could be due to the reticulocytopenia in PRCA patients and/or the transfusion effect of normal red blood cells on PRCA patients. There was no significant difference between LGLL patients with and without PRCA for gender, age, associated diseases, B symptoms, splenomegaly, lymphadenopathy, Hb, ANC, ALC, and platelet count (Table 1).

Among the 34 LGLL patients with isolated anemia, αβ T-LGLL was the most common type, followed by γδ T-LGLL and CLPD-NK, regardless of the presence or absence of PRCA (Table 2). As expected, patients with PRCA were found to have significantly lower bone marrow cellularity with median cellularity of 27.5% compared to 45% in patients without PRCA (*p* = 0.0007), mainly due to markedly reduced to absent erythroid precursors in PRCA patients (Table 2). The percentage of bone marrow involvement by LGLL cells revealed no significant difference between patients with and without PRCA (Table 2). *STAT3* mutation status was available in 8 Caucasian patients, which showed mutations in 3 patients, including Y640F in 1 patient and N647I in 2 patients. *STAT3* mutations were detected exclusively in LGLL patients without PRCA in this small cohort of patients. Similar to the 210 LGLL patients without isolated anemia (data not shown) and the previous reports [23, 24, 29], the 34 LGLL patients with isolated anemia had clonal T-cell or NK-cell populations and an intrasinusoidal distribution of cytotoxic T or NK-cells.

**Table 2:** Pathologic features of LGLL patients with isolated anemia presenting with and without PRCA^a^.

Pathologic characteristic	With PRCA (*n* = 13)	Without PRCA (*n* = 20)	*P* value
Flow cytometric findings			
T-LGLL, αβ	10	15	0.319
T-LGLL, γδ	2	3	0.374
CLPD-NK	1	2	0.452
Bone marrow biopsy			
Median cellularity, %, median	27.5%	45%	0.0007
Erythroid quantity, median	−3^b^	0^b^	<0.00001
Myeloid quantity, median	0^b^	0^b^	0.769
Megakaryocytic quantity, median	0^b^	0^b^	0.196
LGLL involvement, %, median	15%	20%	0.365
Intrasinusoidal cytotoxic cells	10 (77%)	14 (67%)	0.287
TIA-1	9 (69%)	13 (70%)	0.286
Granzyme B	9 (69%)	13 (65%)	0.286
Clonality			
Clonal T cells in T-LGLL	12 (100%)	18 (100%)	1
Clonal NK cells in CLPD-NK	1 (100%)	2 (100%)	1
*STAT3* mutation	0/3 (0%)	3/5 (60%)	0.196

^a^One patient did not have bone marrow biopsy, hence is not included here.

^b^0 normal, −3 markedly decreased.

4 out of 31 patients had cytogenetic abnormalities: 1 PRCA and 1 non-PRCA patient had a loss of the Y chromosome, 1 PRCA patient had a loss of the Y chromosome and a trisomy 15 in a subset of cells tested. In adult males, the absence of a Y chromosome and trisomy 15 without any other abnormality in metaphases from bone marrow is likely age-related and not pathogenic [30, 31]. 1 non-PRCA patient showed del(1),der(3) in 2/20 metaphases, which completely disappeared after T-LGLL was treated, and appeared after T-LGLL relapsed. This result indicates del(1),der(3) represents the T-LGLL clone.

### Response to therapy and clinical outcomes of LGLL patients with isolated anemia presenting with and without PRCA

32 patients were followed for a median duration of 51 months (range: 6–199), including 11 PRCA with a median follow-up of 80 months (range: 6–155) and 21 non-PRCA patients with a median follow-up of 45 months (range: 6–199). All 11 PRCA patients received treatment, while 13/21 (62%) of patients without PRCA received treatment, and the remaining 8 patients were observed. Cyclophosphamide, cyclosporin, or methotrexate was the first-line therapy for those patients. A minimum of 4 months of therapy was required before assessing response. 20 of 24 treated patients exhibited CR (*n* = 14) or PR (*n* = 6) to the first-line therapy with an overall response rate of 83% and a median response duration of 48 months (range: 12–129). Among 14 CR patients, 7 were in CR after cyclophosphamide therapy, 4 after cyclosporin therapy, and 3 after methotrexate therapy. Among 6 PR patients, 4 were in PR after methotrexate therapy, and 2 after cyclophosphamide therapy. The dosing and duration of treatment varied. In general, the dosing was as follows: cyclophosphamide 50 mg to 100 mg PO daily, cyclosporin 25 mg to 200 mg PO daily, methotrexate 7.5 mg to 20 mg PO weekly. The duration of treatment was between 4 and 136 months during follow-up. Cyclophosphamide was usually stopped after a median duration of 12 months of treatment, whereas methotrexate and cyclosporin were used until the last follow-up. Four patients did not respond to cyclophosphamide (*n* = 2) or methotrexate (*n* = 2) as first-line therapy; among the 3 patients who received subsequent lines of treatment, none responded. Steroid therapy was not used as first-line single-regimen therapy but was combined with other first-line agents in 9 (38%) patients (5 with cyclophosphamide, 3 with methotrexate, and 1 with cyclosporin). Four patients received prednisone 5–10 mg daily for 4–12 months; 5 patients started with prednisone 50–120 mg daily with a gradual taper over the ensuing months. Among 14 patients who achieved CR, 8 received steroids in addition to first-line therapy.

**Fig. 1:**
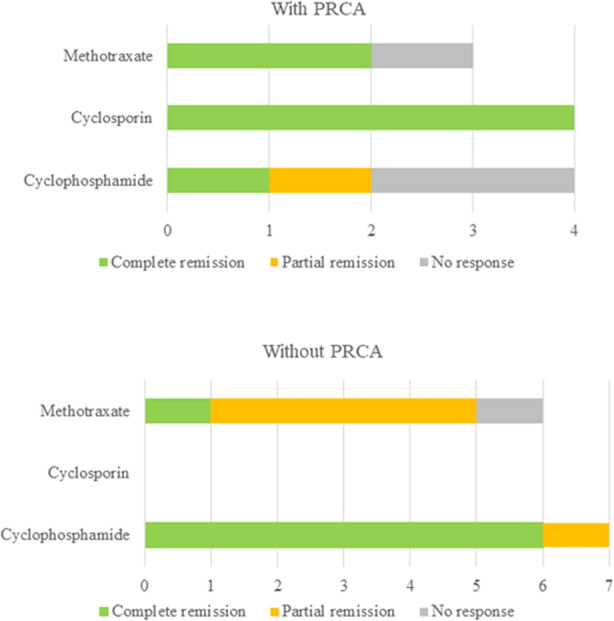
Response to first-line therapy in LGLL patients with isolated anemia presenting with and without PRCA. X-axis represents the number of patients. Green, orange, and gray bars represent complete remission, partial remission, and no response, respectively.

8/11 (72.7%) PRCA patients were in CR (*n* = 7) or PR (*n* = 1) after first-line treatment; 12/13 (92.3%) non-PRCA patients responded to first-line treatment with 7 in CR and 5 in PR. The median response duration was 45 months (range 21–118) for non-PRCA patients and 48 months (range 12–129) for PRCA patients, with no significant difference between the two groups (*p* = 0.989). The type of first-line therapy and overall responses to treatment in patients with and without PRCA were displayed in Table 3. The responses to different first-line therapy administrated to LGLL patients with and without PRCA are illustrated in Fig. 1.

**Table 3:** Clinical outcomes of LGLL patients with isolated anemia presenting with and without PRCA.

Clinical outcomes	With PRCA (*n* = 11)	Without PRCA (*n* = 21)	P
**Follow-up duration, month, median**	75 (6–155)	45 (4–189)	0.366
**Observe (no treatment)**	0 (0%)	8 (38%)	0.029
**Treatment**	11 (100%)	13 (62%)	0.029
**First-line therapy**			
Cyclophosphamide	4 (36%)	7 (54%)	0.228
Methotrexate	3 (27%)	6 (46%)	0.283
Cyclosporin	4 (36%)	0 (0%)	0.009
**Response to first-line therapy**			
Complete remission	7 (64%)	7 (54%)	0.697
Partial remission	1 (9%)	5 (38%)	0.166
No response	3 (27%)	1 (8%)	0.587

Among the 14 CR patients, 6 (3 with PRCA, 3 without PRCA) had a relapse of disease after response to first-line therapy of cyclophosphamide (*n* = 3), cyclosporin (*n* = 2), or methotrexate (*n* = 1), after 1 to 10 years of CR. Three of them were treated subsequentially: one was treated again with cyclophosphamide and achieved CR; two were treated with second-line therapy (cyclosporin was the first-line, cyclophosphamide was the second-line therapy) and both patients achieved PR.

Among the 8 patients without PRCA who were under observation, 6 had follow-up (median:16 months; range: 13-199): 3 patients had progressive anemia (Hb level decreased by >1 g/dL), and 3 showed persistent anemia.

2 of 3 CLPD-NK patients (one with PRCA and the other without PRCA) were in complete remission when treated with methotrexate. The third patient was observed but no follow-up was available.

**Fig. 2:**
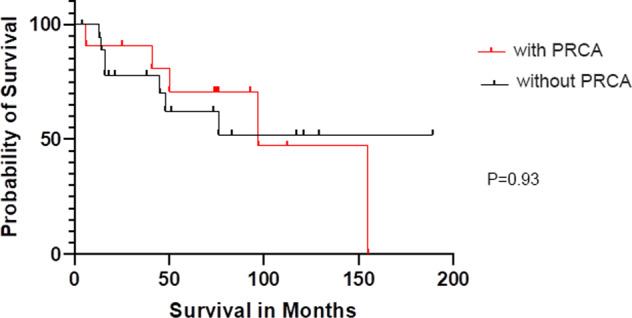
Overall survival of LGLL patients with isolated anemia presenting with and without PRCA. Red line and black line represent patients with PRCA and patients without PRCA, respectively.

Although neutropenia is the most common presentation in LGLL patients, at the end of the follow-up period, no patients developed neutropenia, while 5/27 (18.5%) patients (4 with PRCA, 1 without PRCA) developed thrombocytopenia with a median platelet count at 108 × 10^9^/L (range: 81–117). No significant difference in overall survival (OS) was noted between PRCA and non-PRCA patients (log-rank test *p* = 0.93) (Fig. 2).

## Discussion

Our study demonstrates the importance of recognizing LGLL as a potential cause of isolated anemia, including PRCA and non-PRCA. To the best of our knowledge this is the first report that addresses isolated anemia as a clinical presentation for LGLL patients. Because isolated anemia is a frequent cause for hematologic evaluation, including LGLL as one of the considerations is important for a proper evaluation, diagnosis, and treatment. The reliable clues that necessitate further LGLL workup in patients with isolated anemia include lymphocytosis, morphological LGL in the peripheral blood, and decreased normal NK-cell percentage/number in the peripheral blood as previously reported [29].

Anemia has been reported in 20–85% of LGLL patients, often in bicytopenic or pancytopenic patients [32, 33]. LGLL is rarely considered a cause when patients present with isolated anemia because this relationship has not been well established in the literature. In our cohort of 244 LGLL patients, isolated anemia was present in 34 (14%) patients. For these 34 patients, we excluded other causes as major contributors to the patients’ anemia through extensive workup and proposed LGLL was the underlying mechanism for their anemia. This is further supported by the fact that 20 of 24 treated patients in this cohort responded to the first-line therapy for LGLL (14 in CR and 6 in PR), and half of the observed patients experienced worsening anemia.

Most clinicopathologic features were similar among LGLL patients despite isolated anemia, and they were similar to those of LGLL patients reported by multiple groups [3, 32–39]. Interestingly, LGLL patients with isolated anemia were male predominant (30 of 34, 88%), which was significantly higher than the remaining 210 LGLL patients without isolated anemia (126/210, 60%). Low testosterone levels could cause mild anemia in older men [40, 41]. However, the vast majority of male patients in our study (28/30, 93%) had moderate to severe anemia (median Hb 9.1 g/dL, range 5.2–10.2), making this possibility unlikely. A sample bias could not be excluded, given the relatively small number of cases. Therefore, a larger cohort is required to confirm this finding. Rheumatoid arthritis was significantly less common in isolated anemia patients than those without isolated anemia. This could be related to the absence of neutropenia at diagnosis and during follow-up of LGLL, as neutrophil lysis plays an important role in the immunopathogenesis of rheumatoid arthritis [42].

PRCA is a rare hematologic syndrome characterized by anemia, reticulocytopenia, and severe erythroid hypoplasia of the bone marrow that could be caused by immune conditions, medications, viral infections, thymoma, LGLL, or idiopathy. Approximately 10–30% of PRCA patients have T-LGLL, and 10% of T-LGLL patients have PRCA [19] [43–45]. Among our cohort of 34 LGLL patients with isolated anemia, 38% of patients had PRCA and most did not. Although PRCA patients had markedly decreased reticulocyte count in the peripheral blood and erythroid precursors in the bone marrow and they are more transfusion-dependent, there was no other significant clinicopathologic difference between PRCA and non-PRCA patients.

The discovery of *STAT3* mutations has shed light on the genetic basis of LGLL pathogenesis [26, 46]. Although T-LGLL patients with PRCA have been reported to preferentially harbor *STAT3* mutations [44, 47, 48], none were found in the PRCA patients in this cohort. This is likely due to the small number of cases investigated.

More PRCA patients were given LGLL therapy than non-PRCA patients, likely because PRCA patients were more transfusion-dependent. Among the treated patients, both PRCA and non-PRCA groups exhibited higher therapeutic response rates (72.7% in PRCA and 92.3% in non-PRCA), compared to the reported overall response rate of 40–60% in typical LGLL patients [28] [32, 36, 49]. Our study confirmed the previous finding that LGLL patients with PRCA showed superior response to immunosuppressive therapy [19, 20]. In addition, this is the first study that demonstrates LGLL patients with non-PRCA related isolated anemia also having an excellent therapeutic response to LGLL treatment. Half of the observed non-PRCA patients experienced progressive anemia, which warrants LGLL treatment in the future. This cohort’s high therapeutic response rate (83%) further confirms that LGLL is the etiology for the anemia and emphasizes the importance of LGLL diagnosis in patients with isolated anemia who may benefit from LGLL treatment.

It is uncertain the mechanism of how LGLL causes cytopenia, but the direct cytotoxicity effect on hematopoietic cells is an appealing hypothesis. In patients with PRCA, both in vitro and in vivo data suggested that erythropoiesis at a level of differentiation between early and late erythroid progenitors was inhibited by large granular lymphocytes, which was mediated by direct cell-cell interaction [48, 50]. Immature erythroid elements were essentially absent in PRCA patients but present in non-PRCA patients, suggesting LGLL cells inhibited both immature and mature erythroid elements that might share similar cell surface antigens at successive stages of differentiation. Interestingly, no LGLL patients with isolated anemia developed neutropenia during a median follow-up of 51 months. It is consistent with the previous reports that neutropenia was uncommon in T-LGLL patients with PRCA [18]. The finding raises a possibility that LGLL sometimes might apply lineage-specific inhibition mechanisms, especially in this LGLL group with much lower incidence of rheumatoid arthritis. We recognize that anemia is associated with neutropenia in many LGLL patients, and they may occur at different time points in the course of LGLL [34, 36]. The relatively short median follow-up in this study could not be long enough for the development of neutropenia. Therefore, a longer follow-up may be informative. Nevertheless, it is crucial to recognize that LGLL could cause isolated anemia at initial presentation as these patients may not develop neutropenia until much later.

In conclusion, anemia can uncommonly be the sole cytopenia at the initial diagnosis of LGLL, with PRCA seen in one-third of these patients. Compared to PRCA patients, isolated anemia patients without PRCA demonstrate similar clinicopathologic features, similar therapeutic responses, and comparable overall survival, indicating they are the continuum of the same disorder. This study highlights the importance of recognizing LGLL as a potential cause of isolated anemia at the initial presentation which allows for beneficial LGLL-related treatment.
